# Meteorology and Medical Topography

**Published:** 1868-04

**Authors:** S. D. Richardson


### METEOROLOGY AND MEDICAL TOPOGRAPHY.

BY S. D. RICHARDSON, M. D.

*Mr. President, and Gentlemen of the Iowa State Med. Society:*

At the meeting of this Society one year ago, when I proposed a Committee on Meteorology and Medical Topography, I had a very indefinite idea of the amount of labor necessary for the accumulation of facts in sufficient amount to form the basis of such a report as in my judgment is required, that it may be of use in correcting our ideas and increasing our knowledge of meteorological and endemic influences upon health and disease. I hoped the proposition would elicit remarks from some gentlemen who were somewhat familiar with taking such observations, and therefore would be able to enlighten us upon the practicability of making such a report.

We know that it is accepted by common consent, and conceded by physicians generally, that the influence of the weather is one of the most frequent causes of disease: for nothing is more common than to attribute sickness to having taken *cold.*

Now, to what extent meterological and endemic influences are *really* causes of disease can be ascertained only by accurate observation of all the symptoms of disease at each visit, and the effect of the treatment used, and a faithful registration of the same at the bedside of the patient, ready for comparison with the record of meterological observations at corresponding dates and places.

I think I see in this direction a broad field almost uncultivated, which, if improved by well directed labor, and faithful, patient, and accurately recorded observation, will yield a harvest of truth of great practical value to the physician. But the observations of one man, or any small number of men, however accurate, and although closely compared and studied, can not be productive of a large return.

At some twenty or more places in this State meterological observations are taken daily, and reports made monthly or quarterly to the Smithsonian Institute, Washington, D. C.

These reports show the temperature, the barometric and hydrometric condition of the atmosphere. At the end of the year, I understand, these reports are all published, and copies can be obtained by sending to the proper officers of the Institute.

Mr. President, if you will continue a committee on Meterology and Medical Topography, I believe that by a proper concert of action much valuable practical information can be deduced from such observations, and would suggest the following plan, viz:

Appoint five or more physicians—the more the better—who are in full practice, residing in different and distant parts of the State; each at a place where meterological observations are taken; each of the appointees to be requested to keep a record of the diagnosis of his cases, the symptoms and treatment of the same from day to day, and especially cases of intermittent and remittent fevers, diptheria, typhoid fever, cholera, erysipelas, rheumatism, or any disease of a malignant character, and furnish the chairman of the committee with a copy of the same at least quarterly. Each should also furnish the topography of the district wherein his cases occurred. If this is made a delegated body, composed of delegates from couuty societies, then a report from each county, by a person appointed to make the same, should contribute to make up the report for the State society.

When this practice is generally adopted by medical men it will be easy to collect reports of cases to compare with the meterological reports, and thus furnish the data for a report on Meterology and Medical Topography worthy the attention of our State Society.

